# IS DELIRIUM EDUCATION IMPORTANT: MEDICAL STUDENTS’ ATTITUDES FOLLOWING A DELIRIUM WORKSHOP

**DOI:** 10.1093/geroni/igae098.3353

**Published:** 2024-12-31

**Authors:** Benjamin Rosenstein

**Affiliations:** University of Minnesota, Department of Family Medicine and Community Health, Minneapolis, Minnesota, United States

## Abstract

Delirium is an all too frequent syndrome for which older adults, particularly those with dementia, are at an increased risk. As the population ages, medical students will encounter adults affected by delirium increasingly frequently during their rotations. Unfortunately, education related to delirium is inconsistent within US medical schools. Prior studies have shown students gain knowledge and skills following specific delirium education courses. However, medical students’ attitudes related to delirium as an educational topic have not previously been explored. This study used a thematic analysis to review 4th-year medical students’ reflections of a workshop focused on delirium evaluation, management, treatment, and outcomes. The review revealed 8 primary themes including aspects such as: students frequently encountered older adults experiencing delirium but were unaware of associated poor outcomes, they have limited to any education on delirium during their rotations, they feel this is an often overlooked yet important topic, and they found the workshop provided valuable skills applicable to their current and future practices. Most surprising was medical students’ recognition and reflection of ageist practices they had encountered, though it was not a specific topic within the workshop. This study demonstrates that delirium should be a standard topic in medical education. It provides significant clinical value and may improve medical students’ knowledge, practices, and attitudes, including counteracting ageism, thus promoting age-friendly care of older adults.

